# Immune Mediator Profile in Aqueous Humor Differs in Patients with Primary Acquired Ocular Toxoplasmosis and Recurrent Acute Ocular Toxoplasmosis

**DOI:** 10.1155/2019/9356728

**Published:** 2019-02-17

**Authors:** Claudia Thieme, Stephan Schlickeiser, Sylvia Metzner, Claudia Dames, Uwe Pleyer

**Affiliations:** ^1^Department of Ophthalmology, Charité-Universitätsmedizin Berlin, Corporate Member of Freie Universität Berlin, Humboldt-Universität zu Berlin and Berlin Institute of Health, Germany; ^2^Institute for Medical Immunology, Charité-Universitätsmedizin Berlin, Corporate Member of Freie Universität Berlin, Humboldt-Universität zu Berlin and Berlin Institute of Health, Germany

## Abstract

**Purpose:**

To compare the intraocular cytokine and chemokine profiles in patients with acute primary acquired ocular toxoplasmosis (pOT) or recurrent ocular toxoplasmosis (rOT) and to correlate them with their clinical characteristics.

**Methods:**

Aqueous humor samples were collected from 62 consecutive patients (21 pOT, 30 rOT, and 11 noninfected controls) and analyzed by multiplex assay. Correlations were assessed between cytokine/chemokine levels, type of inflammatory response (T_h_1, T_h_2, and T_h_17), and clinical characteristics. In all OT patients, the clinical diagnosis of either pOT or rOT was confirmed by positive intraocular Goldmann/Witmer-Desmonts coefficient. Correlations were assessed between a preselected panel of immune mediators and the clinical characteristics of OT.

**Results:**

In pOT patients, increased levels of IL-2, IFN-*γ*, TNF-*α*, IL-15, IL-4, IL-5, IL-9, IL-13, IL-17, IL-1R*α*, IL-6, IL-1*β*, and chemokines MIP-1*α*, MIP-1*β*, IP-10, Eotaxin, IL-8, RANTES, PDGF-bb, GM-CSF, G-CSF, and MCP-1 were found in comparison to those in controls (*p* < 0.05). Patients with rOT showed elevated levels of IL-2, IFN-*γ*, TNF-*α*, IL-15, IL-4, IL-5, IL-9, IL-17, IL-1R*α*, IL-6, IL-1*β*, and chemokines MIP-1*α*, IP-10, Eotaxin, IL-8, RANTES, PDGF-bb, G-CSF, and MCP-1 compared to controls (*p* < 0.05). In addition, IL-7 (*p* = 0.028) differed between pOT and rOT; IL-9 (*p* = 0.054) and IL-13 (*p* = 0.051) showed a tendency of higher concentration in pOT than in rOT. A negative correlation was found between IL-7 (*p* = 0.017) as well as IL-9 (*p* = 0.008) and the number of recurrences. Cytokine ratios showed no difference between pOT and rOT, indicating a dominant T_h_1-type response in both infectious groups. Moreover, a positive correlation was detected between IL-7, VEGF, IL-13 and age at aqueous humor sampling (*p* < 0.05).

**Conclusions:**

This study for the first time shows subtle differences between the intraocular cytokine profiles in patients with either acute pOT or rOT.

## 1. Introduction

The protozoan *Toxoplasma gondii* (*T. gondii*) establishes a lifelong chronic infection that targets neuronal tissues such as the brain and the retina. Ocular toxoplasmosis (OT) remains one of the leading causes of visual impairment and is the most frequent cause of infectious posterior uveitis [1]. It typically presents with focal inflammation and necrosis of the neurosensory retina, affecting retinal glial and pigment epithelial cells [2]. The severity of OT varies mainly due to the genotype of the pathogen, the genetic background of the patient and its immune response. The genotype of the pathogen has been recognized for some time, as more virulent strains with pronounced clinical manifestations are observed, in particular in South America compared to Europe [1, 3]. This impacts the immune response and clinical course of *T. gondii* infections. During the acute stage of the infection, an intraocular IFN-*γ*-mediated immune response and, to a lesser degree, humoral immunity limit parasite growth induced conversion of tachyzoites to dormant bradyzoites. But the parasite eventually establishes a chronic infection leading to a lifelong risk of exacerbation with significant threat for visual impairment and psychological burden [4].

While the physiopathological mechanisms that lead to reactivation of OT are not yet fully understood, the intraocular immune response plays an essential role [5]. Subsequently, mapping of intraocular mediators has become a field of major interest since it may provide better guidance for more targeted treatment and prognostic outcome. Thus, a predominant T_h_2 response has been related to a more severe clinical manifestation in a cohort of 9 Columbian patients. In the same study, IL-5 and VEGF were associated with more frequent recurring OT [6]. In contrast, high levels of IFN-*γ*, IL-6 and MIP-1 indicating a strong T_h_1 immune response have been consistently reported in Europe. Taken together, a first “fingerprint” of the immune response is emerging and potentially may serve as a “biomarker” with further clinical importance [6]. Nevertheless, contradictory results persist, which are related to (a) differences in the OT strain—host interaction—(b) the stage of the ocular disease, and (c) often the insufficient number of patients. In particular, to our knowledge, there exists no reliable information regarding the distinction between the initial manifestation and further occurring reactivation of OT. Although reactivation of OT is considered as an important problem, none of the previous studies have differentiated between acute primary (pOT) and recurrent OT (rOT) in European patients. So far, there is virtually no data available differentiating between the intraocular milieu in patients with acute pOT and rOT. It has been suggested that the intraocular cytokinome provides a rationale to delineate potential options to intervene and prevent further disease progression.

Therefore, here, we evaluated the intraocular immune response in patients with active pOT and rOT. We not only investigated whether there is a difference in these two clinical cohorts but also correlated the intraocular expression of inflammatory mediators with clinical findings. Our results indicate for the first time that the cytokinome differs in active pOT and rOT and may open further avenues for guidance on severity and risk of recurrence in this infection.

## 2. Methods

### 2.1. Study Populations

All 51 OT patients and 11 controls were seen by an experienced ophthalmologist (UP). They underwent a complete ocular exam including best corrected Snellen visual acuity, slit lamp biomicroscopy, tonometry, indirect ophthalmoscopy, OCT and visual field testing. The diagnosis of OT was (a) based on retina visualization by binocular ophthalmoscopy with a +20-diopter lens (Volk Corporation, USA) and (b) secured by biological testing of intraocular fluids as previously reported [7, 8].

All patients were seen between December 2005 and April 2014 at Charité-Universitätsmedizin Berlin, Department of Ophthalmology, a tertiary referral and uveitis center. All subjects were immunocompetent without a past medical history of immune modulatory or cytotoxic agents. Since patient age has been discussed as a confounding factor in OT recurrences, we arbitrary allocated patients being either younger or older than 40 years.

#### 2.1.1. Patients with Acute Primary Ocular Toxoplasmosis

Acute pOT was defined as the presence of an active lesion (creamy white, focal retinal lesion) not associated with retinochoroidal scars in either eye following the internationally accepted diagnostic criteria [9].

#### 2.1.2. Patients with Acute Recurrent Ocular Toxoplasmosis

Acute rOT was defined as the presence of such an active lesion and at least one hyperpigmented retinochoroidal scar formation due to OT [9]. Episodes of anterior segment inflammation in the eyes with retinochoroidal scars without active retina lesions were not considered as recurrences and excluded. There was only one patient with congenital acquired OT.

#### 2.1.3. Control Group

The control group consisted of 11 patients who had undergone routine cataract surgery. These individuals had no preexisting ocular disease, except cataract; in particular, they had no lesion suspicious for previous intraocular infection, nor previous ocular surgery. Samples of aqueous humor were obtained initially at cataract surgery and proved negative for intraocular *T. gondii* antibody synthesis.

### 2.2. Clinical Evaluation and Intraocular Inflammation Assessment

In all patients, we graded the level of inflammation based on the presence of cells in the anterior chamber and/or vitreous haze (grade 0-IV) according to the criteria proposed by the International Uveitis Study Group (IUSG) and Standardization of Uveitis Nomenclature (SUN) [10]. To document the location and size of retina lesions, fundus photographs were taken by a Zeiss Fundus camera FF 450 plus (Zeiss, Jena, Germany) and the size of retinochoroidal lesions was measured in optic disc diameters.

### 2.3. Sample Collection and Processing

Following informed consent, aqueous humor sampling was undertaken under topical anesthesia. All procedures were performed under aseptic conditions in an ocular surgery setting. Briefly, a 31-gauge needle was inserted at the peripheral clear cornea and between 100 and 300 *μ*l aqueous humor could be withdrawn under direct control of an operating microscope [8]. All samples were immediately stored and maintained at -80°C to prevent degradation and thawed directly before analysis.

### 2.4. Measurement of Immune Mediators in Aqueous Humor Samples

The Bio-Plex Pro Human Cytokine 27-Plex Immunoassay (Bio-Rad) was used to measure concentrations of immune mediators in the aqueous humor samples. The immune mediators were classified into 8 categories: the T cell development-promoting cytokines, T_h_1-derived cytokines, T_h_1 cell development-promoting cytokines, T_h_2-derived cytokines, T_h_17-derived cytokines, T_h_17 cell development-promoting cytokines, chemokines, and growth, angiogenetic and wound-healing factors.

The layout of the assay consisted of eight standards in duplicate, two blank wells, and 62 aqueous humor samples. Levels of immune mediators were calculated in pg/ml using standard curves of known concentrations. The data were analyzed with Bio-Plex Manager software, version 1.1. All measurement values were extrapolated beyond standard ranges to calculate concentrations by 5-parameter logistic regression, and these were used for statistical comparisons by nonparametric testing.

### 2.5. Ethical Considerations

The study followed the tenets of the Declaration of Helsinki and was approved by the institutional ethics committee of Charité. All patients provided written informed consent and the institutional review board of ethics approved this study (registration number 8/6109).

### 2.6. Statistical Analysis

The software GraphPad Prism Version 7.04 was used for the statistical analysis to compare immune mediator levels of the three groups (pOT, rOT, and controls) by Kruskal-Wallis test followed by uncorrected Dunn's test. We did not correct for multiple comparisons in order to not inflate type II errors in this exploratory study. To detect correlations between clinical parameters and immune mediator levels, Spearman rank correlation was applied. Since ratios of immune mediators, e.g. the TNF-*α*/IL-10 relation, have been previously supportive in determining the significance of particular pathways, the following cytokine ratios were formed: IFN-*γ*/IL-4, IFN-*γ*/IL-10, IL-17/IFN-*γ*, TNF-*α*/IL-10, and IL-12p70/IL-10. We added one to all cytokine values relevant for the cytokine ratio in order to avoid zero values.

The Poisson test was used for the calculation of the risk of recurrence using IBM SPSS Statistics Version 19. Boxplots and graphs were created with the software GraphPad Prism Version 7.04 [11]. To analyze the frequency of age distribution between groups, the chi-square test was performed [12]. A statistically significant result was defined as *p* ≤ 0.05.

## 3. Results

### 3.1. Patient Demographics

We enrolled 51 individuals with clinical characteristics of OT and additional serological intraocular confirmation of the infection. Based on the predefined diagnostic criteria, 21 patients presented with pOT, whereas 30 individuals suffered from rOT [9]. The mean age at the first episode of ocular infection was 39 ± 15 years in the pOT group and 30 ± 15 years in the rOT group. The age at aqueous humor sampling was 39 ± 15 years in the pOT group and 38 ± 15 years in the rOT group. No difference in age was present in both OT cohorts, but as can be expected, individuals undergoing cataract surgery (controls) were older (mean age: 75 ± 7 years; *p* < 0.001, Table 1). The rate of recurrences was more than 2 times higher in patients less than 40 years of age (Table 1).

### 3.2. Clinical Findings and Grading of Intraocular Inflammation

The clinical characteristics of all participants are summarized in Table 1. Briefly, all OT patients presented typically with active retinochoroiditis with whitish, moderately exudative, ill-defined retina lesions (100%) [1, 9]. Of interest, bilateral OT was rare and exclusively seen in few (*n* = 6) patients with recurrent ocular lesions. However, at no time, simultaneous inflammatory activity was observed.

Inflammation in the anterior chamber ranged from 0 to +2 in both OT groups and did not differ significantly in pOT (median grade: 0) compared to rOT (median grade: 0), even when pOT patients were more often affected (32% vs. 24% in rOT). Similarly, vitreous haze was not only more frequently seen in pOT (46% vs. 24% in rOT) but also appeared as more intense at initial infection (pOT: grade 1-3 as compared to rOT: grade 0.8-2). Both findings, however, did not prove to be significant. Finally, we documented a wide range in the size of retina lesions, which extended for up to 8 disc diameters but we were unable to detect a statistically significant difference between groups (pOT vs. rOT, *p* > 0.05).

### 3.3. Comparison of Immune Mediator Concentrations in pOT, rOT, and Controls

**Figure 1:**
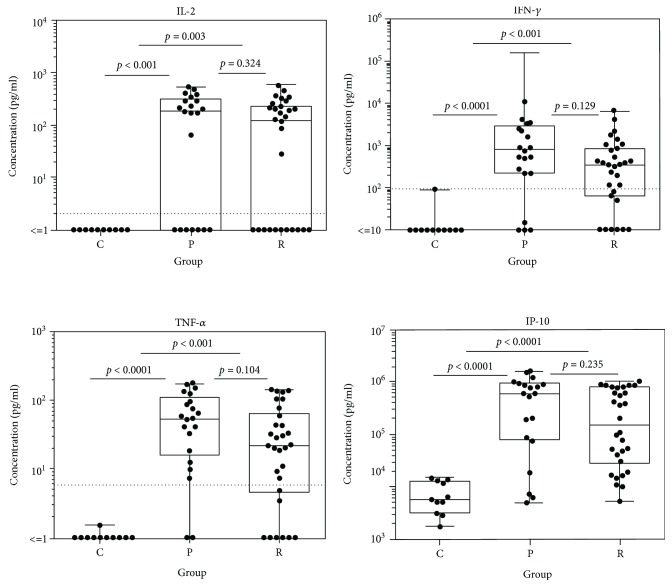
Box plot depicting the profile of the T cell development-promoting cytokine IL-2 and T_h_1-derived cytokines IFN-*γ* and TNF-*α* and the chemokine IP-10 in aqueous humor of patients with pOT (*n* = 21) and rOT (*n* = 30) and the control group (*n* = 11).

**Figure 2:**
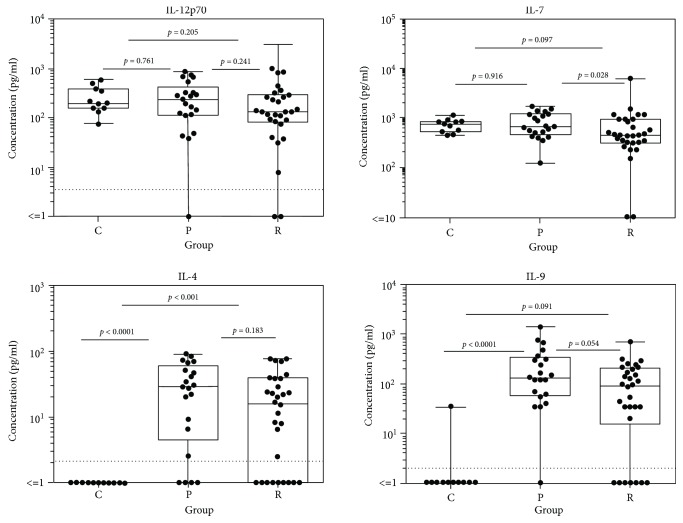
Box plots depicting the cytokine patterns of T_h_1 cell development-promoting cytokines IL-12p70 and IL-7 and the T_h_2-derived cytokines IL-4 and IL-9 in aqueous humor comparing results of patients with pOT (*n* = 21) and rOT (*n* = 30) and the control group (*n* = 11).

**Figure 3:**
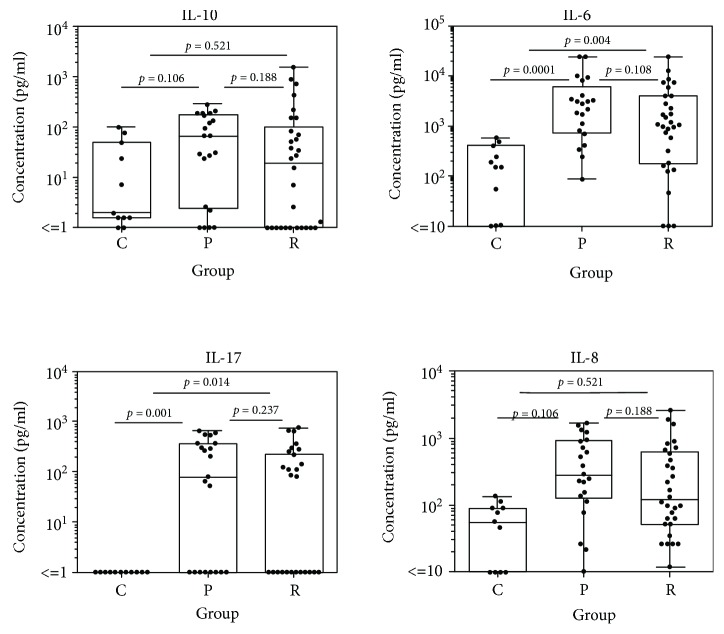
Cytokine patterns of the T_h_2 cytokine IL-10, the T_h_17 cell development-promoting cytokine IL-6, the T_h_17-derived cytokine IL-17, and the chemokine IL-8 comparing immune mediator concentrations in aqueous humor of patients with pOT (*n* = 21) and rOT (*n* = 30) and the control group (*n* = 11).

A panel of immune mediators was significantly upregulated in patients affected by acute pOT. This includes cytokines of the T_h_1 response. Tables 2 and 3 summarize the data of all inflammatory mediators detected in the aqueous humor of all three cohorts. Comparing pOT and controls, the following immune mediators were significantly elevated: IL-2 (*p* < 0.001), IFN-*γ* (*p* < 0.0001), TNF-*α* (*p* < 0.00001), IL-15 (*p* < 0.0001), IL-4 (*p* < 0.0001), IL-5 (*p* < 0.0001), IL-9 (*p* < 0.00001), IL-13 (*p* = 0.022), IL-17 (*p* = 0.001), IL-1R*α* (*p* < 0.001), IL-6 (*p* < 0.0001), IL-1*β* (*p* = 0.003), MIP-1*α* (*p* < 0.001), MIP-1*β* (*p* = 0.023), IP-10 (*p* < 0.00001), Eotaxin (*p* < 0.001), IL-8 (*p* < 0.001), RANTES (*p* < 0.0001), PDGF-bb (*p* < 0.001), GM-CSF (*p* = 0.036), G-CSF (*p* = 0.004), and MCP-1 (*p* < 0.001). When we analyzed and compared rOT and controls, the following spectrum appeared as given in detail: IL-2 (*p* = 0.003), IFN-*γ* (*p* < 0.001), TNF-*α* (*p* < 0.001), IL-15 (*p* = 0.004), IL-4 (*p* < 0.001), IL-5 (*p* < 0.001), IL-9 (*p* = 0.001), IL-17 (*p* = 0.014), IL-1R*α* (*p* = 0.002), IL-6 (*p* = 0.004), IL-1*β* (*p* = 0.032), MIP-1*α* (*p* < 0.001), IP-10 (*p* < 0.0001), Eotaxin (*p* = 0.007), IL-8 (*p* = 0.009), RANTES (*p* < 0.001), PDGF-bb (*p* = 0.001), G-CSF (*p* = 0.034), and MCP-1 (*p* = 0.009) (Figures 1-3 and Supplementary Figures 1-3). When both groups of infected patients were compared (pOT vs. rOT), we detected IL-7 as elevated in pOT (*p* = 0.028) (Table 2, Figure 2). IL-9 (*p* = 0.054) and IL-13 (*p* = 0.051) showed a tendency of being elevated in pOT when comparing both infectious groups (Table 2).

Since the balance between pro- and anti-inflammatory mediators is important in OT, we calculated the ratios of key regulators. All cytokine ratios showed no significant difference between pOT and rOT, indicating a dominant T_h_1-type response in both infectious groups: IFN-*γ*/IL-4 (*p* = 0.054), IFN-*γ*/IL-10 (*p* = 0.405), IL-17/IFN-*γ* (*p* = 0.499), TNF-*α*/IL-10 (*p* = 0.783), and IL-12p70/IL-10 (*p* = 0.291) did not reveal any shift of the immune response between pOT and rOT (Table 4).

### 3.4. Correlations between Immune Mediator Concentration and Clinical Characteristics

As a second goal, we analyzed whether there is a correlation between expression of immune mediators and clinical manifestations. Even when inflammation was remarkable and appeared even more intense in our pOT individuals, we were not able to correlate intraocular findings, e.g. vitreous haze, with a particular pattern of inflammatory mediators. Similarly, we could not correlate the OT lesion size with any intraocular immune mediator (*p* > 0.05). Whereas this is in line with previous observations in European studies, further findings relate to our major interest, the association of OT recurrences to other variables.

**Figure 4:**
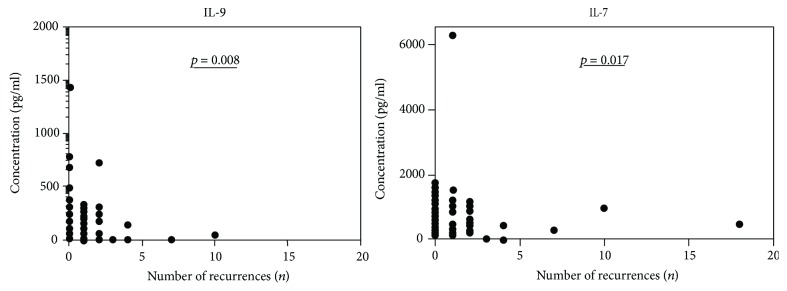
Correlation between cytokine levels and number of recurrences for IL-9 (*p* = 0.008) and IL-7 (*p* = 0.017) in aqueous humor of patients with pOT and rOT (*n* = 51). A value of *p* ≤ 0.05 is defined as statistically significant (Spearman correlation).

Interestingly, a negative correlation between IL-9 (*p* = 0.008), IL-7 (*p* = 0.017) and number of OT recurrences could be detected (Figure 4). The concentration of several other immune mediators was reduced with the number of OT recurrences (Supplementary Table 1). In particular, there were two patients with 18 and 10 OT recurrences who showed an overall reduced immune mediator concentration.

In addition, our findings further indicate a positive correlation between age at consultation and concentration of IL-7 (*p* = 0.018), VEGF (*p* = 0.031) and IL-13 (*p* = 0.030).

**Figure 5:**
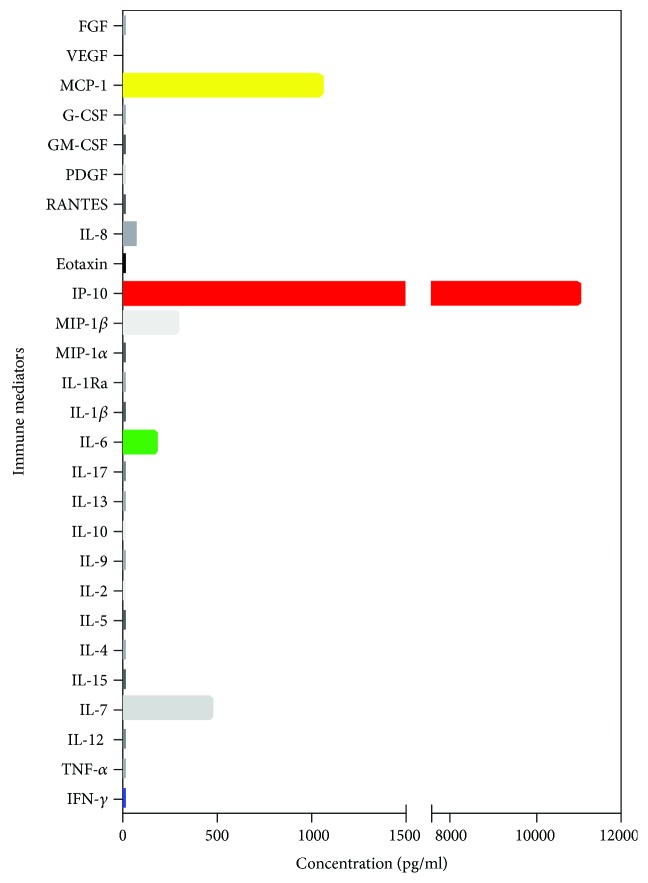
Bar chart showing the pattern of immune mediators in aqueous humor of a patient who had 18 recurrences with generally low immune mediator levels in aqueous humor and elevated levels of IP-10, and concentrations of MCP-1, MIP-1*β*, IL-7, and IL-6 are detectable.

**Figure 6:**
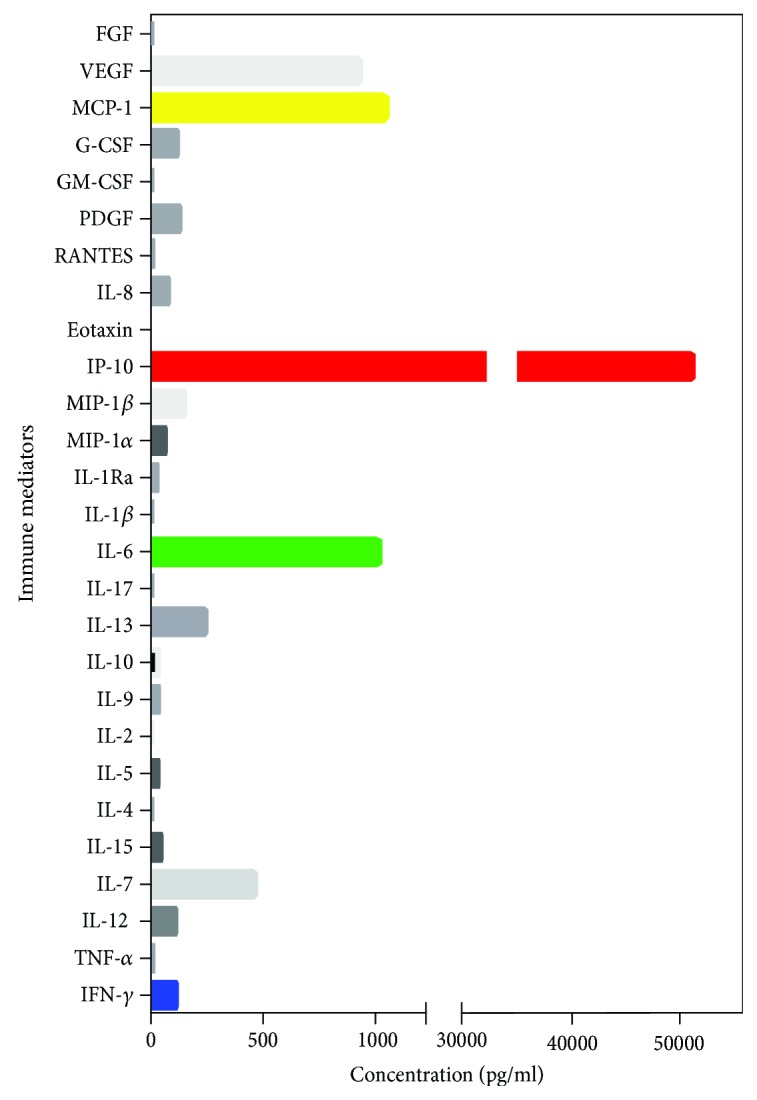
Bar graph showing the profile of immune mediators in aqueous humor of a patient with congenital recurrent OT who had 2 recurrences with low IFN-*γ* levels, but high levels of IP-10, IL-6, MCP-1, and VEGF.

As noted earlier, in our rOT group, patients younger than 40 years of age experienced a higher number of recurrences when compared to individuals above the 4^th^ life decade (patients < 40 years of age: mean = 3.6 ± 4.4 recurrences, median = 2 recurrences; patients > 40 years of age: mean = 2.1 ± 2.8 recurrences, median = 1 recurrences, *p* = 0.032). According to the incidence rate ratio, patients < 40 years of age bear a 2.34 times higher risk for recurrence than patients > 40 years of age (Tables 1, 95% CI 1.38-4.11, *p* = 0.007). Consequently, we were able to calculate the recurrence rate in the group aged below 40 years as 0.385 per person per year, whereas the recurrence rate in the older group dropped to 0.164 per person per year. Two patients of our cohort show exemplarily the reduction of immune mediator levels in aqueous humor: a 28-year old male patient who had 18 OT recurrences with extensive immune mediator reduction (Figure 5) and a 33-year old female patient with congenital OT who had 2 OT recurrences and revealed low IFN-*γ* levels, still with high concentrations of IP-10, IL-6, MCP-1, and VEGF (Figure 6).

## 4. Discussion

Aqueous humor is important for the homeostasis of the eye, and it reflects the “milieu interior.” Therefore, it is used in diagnosis and to investigate immune-mediated changes [13, 14]. Although recovery of aqueous humor is not a harmful procedure, it is not routinely performed in most uveitis centers. Thus, currently, only very limited information is available on intraocular cytokine profiles in patients with OT.

In this study, we compared the intraocular cytokine profiles of acute pOT and rOT for the first time. Increased levels of IFN-*γ*, TNF-*α*, IL-4, IL-17, IL-6, MIP-1*α*, MIP-1*β*, IP-10, IL-8, RANTES, and MCP-1 were detected in both primary and recurrent OT compared to controls. Interestingly, immune mediator release was reduced with the number of recurrences.

The immune response in active OT is initiated by the recognition of the parasite, which stimulates the production of cytokines such as IFN-*γ* and TNF-*α* by cells including NK cells and macrophages [15, 16]. These cells and mediators control local inflammation and dissemination of tachyzoites. Furthermore, a T_h_1-cell-dominated adaptive immune response rapidly controls the dividing tachyzoite stage of *T. gondii* [17]. Indeed, in our patients with pOT and rOT, we confirmed a dominant T_h_1-type response with increased expression of IFN-*γ* and TNF-*α*. Both cytokines are produced through resident and immune cells and act synergistically by inhibiting *T. gondii* replication in retinal pigment epithelial (RPE) cells. However, only IFN-*γ* suppresses the parasite within retinal glial cells and it remains undoubtedly the dominant player in antitoxoplasmic immunity [2]. If IFN-*γ* is absent, the risk for recurrent exacerbations of the infection is increased [18, 19].

TNF-*α* contributes to upregulation of IFN-*γ*, and in an experimental mouse model, it played an important protective role in *T. gondii* infection [20]. In our patients, levels of TNF-*α* were more significantly elevated in the first OT episode. IL-6, another cytokine that promotes IFN-*γ* upregulation, was also highly expressed in pOT and, to a lesser extent, in rOT. IL-6 has pleiotropic effects that can be both protective and deleterious during *T. gondii* infection and be pathologic in OT [18, 21, 22]. It leads to significant local recruitment of neutrophils, monocytes and T lymphocytes with destructive effects on retinal architecture [21]. In addition, it downregulates transforming growth factor-*β*, a cytokine that plays a key role in maintaining ocular immune privilege [23, 24] and contributes to induction of intraocular IL-17, another proinflammatory cytokine for which both protective and destructive effects have been reported [25–28]. It has been suggested that increased IL-17 could be related to the often more favorable clinical course in European patients versus South Americans [25]. Two studies examining intraocular cytokines in French patients revealed T_h_1- and T_h_17-type responses, whereas de-la-Torre et al. [25] found that Columbian patients showed predominantly T_h_2-type immune responses, mainly related to divergent *T. gondii* genotypes [25, 26]. Our findings were similar to those in the French patients, with dominant T_h_1- and T_h_17-type responses [20, 22].

Although the IFN-*γ*-mediated immune response is dominant in OT, T_h_2-type cytokines such as IL-4 and IL-13 can also be protective [29]. The balance of IFN-*γ* with anti-inflammatory mediators such as IL-10 is also important [30]. Compared to the control group, we found elevated aqueous humor levels of IL-4 in pOT and rOT and of IL-13 in pOT. However, the T_h_1-type response seems to be dominant in pOT and rOT, as shown by the cytokine ratios (Table 4). We have also shown for the first time in pOT and rOT the intraocular presence of IL-9, a cytokine produced by T_h_2, T_h_9, and T_h_17 cells. There was a tendency to more elevated IL-9 and IL-13 in pOT than in rOT (Table 2), suggesting the possibility that the intraocular absence of these cytokines may play a role in OT recurrence. IL-9 plays a role in various parasite infections [31], and the role of IL-9 in protection against *T. gondii* merits further investigation.

Chemokines play a major role in OT, selectively recruiting monocytes, neutrophils and lymphocytes [32, 33]. CXCL10 had very high levels in both our OT cohorts. Also known as IP-10, it is mainly secreted by monocytes, endothelial cells and fibroblasts, attracts monocytes/macrophages, T cells, NK cells, and dendritic cells, and upregulates the T_h_1-type immune response. In addition, we found increased levels of CCL2 (MCP-1), CCL3 (MIP1-*α*), and RANTES in all OT patients.

An obvious question is whether the immune mediator profile correlates with clinical features. Whereas clinical findings such as size of retinal lesions, extent of inflammation, and vitreous infiltration could not be correlated to any cytokinome profile, the profile in rOT might be of clinical relevance. In our study, the concentration of IL-7 in aqueous humor was low in patients with rOT, with an additional trend towards low IL-9 and IL-13, which may suggest a role for those cytokines in preventing recurrence. A prospective study evaluating the concentrations of cytokines over the course of OT may clarify whether low levels of these cytokines could predict rOT.

Our study found a higher number of recurrences in the overall cohort compared to a recent study in Columbia by de-la-Torre et al. [6]. In particular, the number of recurrences in our study cohort was more than 2-fold higher in our patients younger than 40 years. The results of another study with European patients corresponded with ours, revealing that younger OT patients have a higher risk of recurrences than older OT patients [34]. Contrary to this, Holland et al. [35] found that patients > 40 years of age have a higher risk of recurrence than younger patients. The average age of our entire study cohort was slightly higher compared to that of de-la-Torre et al. [6]; however, the age of our rOT patients was comparable to previous reports [1, 6]. Whereas age did not prove as a confounding factor of intraocular immune mediator expression in previous studies, its role for OT recurrences is still under debate [24, 36]. Interestingly, our patients < 40 years of age had a higher number of recurrences and a higher risk of recurrences (Table 1). It is likely that *T. gondii* attempts to skew the local immune response further down in order to evade the host response and favor its survival and replication.

Whereas the intraocular cytokinome could not be correlated with clinical features, VEGF levels increased with age at aqueous humor sampling in our study cohort. VEGF was previously found to correlate with inactive OT and was suggested to play a role in the formation of neovascular membranes following *T. gondii* infection [6].

Taking into account regional differences, large cohorts from different regions of the world might be necessary to extend our observations on differences between pOT and rOT. The genotype of the pathogen was not analyzed in detail in our study; however, we previously identified type II as dominant in our patients [3]. The current investigation is limited by the cross-sectional design, in which all subjects had aqueous humor sampling during active disease. Although the acute response is probably most important to guide the subsequent clinical course of ocular infection, a follow-up study with aqueous samplings over the course of OT may give us more insightful information regarding the pathogenesis of the disease. We did not sample aqueous humor from OT patients during the quiescent stage of infection for ethical reasons. However, in future studies, it would be valuable to analyze the aqueous humor of OT individuals who need to undergo surgery for other reasons, e.g., the development of cataracts. Similarly, it would be ideal to sample aqueous humor from a larger cohort of healthy individuals with no ocular pathology to serve as controls and it should be stressed that the findings in this exploratory study need to be validated in new cohorts of samples.

In summary, our study indicates that the cytokinome in pOT and rOT has subtle differences and that immune mediator levels in aqueous humor decline with recurrences. Moreover, young patients in our cohort had a higher risk of recurrences for OT. Mapping of mediators may therefore not only strengthen our understanding of the intraocular pathophysiology of this sight-threatening infection but provide guidance on more specific treatment options and prognostic evaluation.

## 5. Summary

Whereas a strong T_h_1-type-mediated immune response dominated in primary ocular toxoplasmosis and recurrent ocular toxoplasmosis, subtle differences in a number of immune mediators could be found between both cohorts.

## Figures and Tables

**Table 1:** Clinical characteristics of the patient in the primary and recurrent OT groups and control group.

	Primary OT	Recurrent OT	Control group	*p* value^a^
Number of patients (*n*)	21/62 (33.9%)	30/62 (48.4%)	11/62 (17.7%)	
Gender	Male: 42.9% (9/21)	Male: 36.6% (11/30)	Male: 63.6% (7/11)	
Interval between consultation and aqueous humor sampling (days)	2 ± 2	3 ± 4		
Observation time after aqueous humor sampling (days)	143 ± 250	577 ± 852		
Mean age at first episode (years ± SD)	39 ± 15	30 ± 15		**p** = 0.043
Mean age at aqueous humor sampling (years ± SD)	39 ± 15	38 ± 15	75 ± 7	pOT/rOT vs control: **p** < 0.001, pOT vs rOT: *p* = 0.821
		Age < 40 years: 18 patients		
		Age > 40 years: 12 patients		
Median grading of inflammation in vitreous humor (grade 0-4, min.-max.)	1 (1-3)	0.8 (0.8-2)		
Median grading of inflammation in the anterior chamber (grade 0-4, min.-max.)	0 (0-2)	0 (0-3)		
Median number of active lesions (*n*, min.-max.)	1 (1-1)	1 (1-4)		
Median size of active lesions (PD, min.-max.)	1 (0-3)	0.3 (0-8)		*p* > 0.05
Median number of scars (*n*, min.-max.)	0	1 (1-7)		
Bilateral OT involvement	0	20% (6/30)		
Median number of recurrent active episodes (*n*)	∅	1.5 (1-18)		
		Age < 40 years: 2 recurrences		
		Age > 40 years: 1 recurrence		**p** = 0.032
Macular involvement	9.5% (2/21)	13.3% (4/30)		
Risk of recurrences		Age < 40 years: 2.34 times higher than >40 years		**p** = 0.007

A summary of the patients' demographics and clinical characteristics in the primary and recurrent OT groups and control group. OT: ocular toxoplasmosis; SD: standard deviation; PD: diameter of the papilla; ^a^ *p* value < 0.05 is considered as statistically significant; significant *p* values are shown in bold.

**Table 2:** Chemokine, growth factor, angiogenetic factor, and wound healing factor concentration in aqueous humor of a control group and patients with pOT and rOT (*n* = 62).

Immune mediator	Mdn concentration in patients with pOT, *n* = 21 (IQR) (pg/ml)	Mdn concentration in patients with rOT, *n* = 30 (IQR) (pg/ml)	Mdn concentration in patients in the control group, *n* = 11 (IQR) (pg/ml)	pOT vs control **p** value	rOT vs control **p** value	pOT vs rOT **p** **value**
*T cell development-promoting cytokine*						
IL-2	183 (0, 315)	123 (0, 233)	*0 (0, 0)*	**<0.001**	**0.003**	0.324
Th1-cytokines						
IFN-*γ*	760 (218, 2.93^∗^10^3^)	348 (62.7, 909)	*0 (0, 0)*	**<0.0001**	**<0.001**	0.129
TNF-*α*	55.2 (15.6, 111.4)	22.6 (4.53, 64.9)	*0 (0, 0)*	**<0.00001**	**<0.001**	0.104
*T_h_1 cell development-promoting cytokines*						
IL-12p70	233 (114, 423)	132 (81.2, 289)	192 (158, 384)	0.761	0.205	0.241
IL-7	654 (464, 1.19^∗^10^3^)	450 (318, 937)	772 (532, 851)	0.916	0.097	**0.028**
IL-15	79.8 (39.9, 217)	47.5 (12.5, 136.5)	*0* (*0*, 23.4)	**<0.0001**	**0.004**	0.084
*T_h_2 cytokines*						
IL-4	29.4 (4.48, 59.6)	16.2 (0, 38.6)	*0 (0, 0)*	**<0.0001**	**<0.001**	0.183
IL-5	49.2 (22.8, 149)	37.1 (15.5, 93.6)	*0 (0, 0)*	**<0.0001**	**<0.001**	0.380
IL-9	135 (58.7, 345)	93.5 (14.9, 205)	*0 (0, 0.62)*	**<0.00001**	**0.001**	0.054
IL-10	67.9 (2.43, 176)	19.8 (0, 102)	1.99 (1.59, 49.8)	0.106	0.521	0.188
IL-13	345.4 (204, 1.05^∗^10^3^)	194 (77.9, 647)	230 (103, 286)	**0.022**	0.402	0.051
*T_h_17 cytokines*						
IL-17	79.9 (*0*, 371)	*0 (0, 234)*	*0 (0, 0)*	**0.001**	**0.014**	0.237
IL-1R*α*	262 (53.5, 648)	130 (0, 337)	*0 (0, 0)*	**<0.0001**	**0.002**	0.207
*T_h_17 cell development-promoting cytokines*						
IL-6	2.74∗10^3^ (738, 6.01∗10^3^)	1.06∗10^3^ (173, 3.99∗10^3^)	150 (1.54, 409)	**<0.0001**	**0.004**	0.108
IL-1*β*	3.96 (*0.57*, 8.11)	*1.38 (0,* 5.36)	*0 (0, 1.49)*	**0.003**	**0.032**	0.220

pOT: primary OT; rOT: recurrent OT: Mdn: median; IQR: interquartile range; significant *p* values are shown in bold; italic values are below LLOQ as defined by the manufacturer.

**Table 3:** Chemokines, growth factor, angiogenetic factor, and wound healing factor concentrations in aqueous humor of a control group and patients with pOT and rOT (*n* = 62).

Immune mediator	Mdn concentration in patients with pOT, *n* = 21 (IQR) (pg/ml)	Mdn concentration in patients with rOT, *n* = 30 (IQR) (pg/ml)	Mdn concentration in patients in the control group, *n* = 11 (IQR) (pg/ml)	pOT vs control *p* value	rOT vs control *p* value	pOT vs rOT *p* value
*Chemokines*						
MIP-1*α*	271 (134, 412)	157 (56.7, 451)	*0* (*0*, 41.2)	<0.0001	<0.001	0.331
MIP-1*β*	396 (299, 604)	283 (178, 593)	218 (166, 341)	0.023	0.210	0.153
IP-10	5.97∗10^5^ (7.93∗10^4^, 9.38∗10^5^)	1.52∗10^5^ (2.75∗10^4^, 7.99∗10^5^)	5.75∗10^3^ (3.17∗10^3^, 13.0∗10^4^)	<0.00001	<0.0001	0.235
Eotaxin	925 (241, 1.32∗10^3^)	755 (0, 1.01∗10^3^)	*1.95 (0,* 290)	<0.001	0.007	0.302
IL-8	280 (128, 937)	120 (51.6, 611)	56.5 (*0*, 90.2)	**<0.001**	**0.009**	0.166
RANTES	219 (61.3, 296)	91.1 (7.4, 372)	*0 (0, 0)*	**<0.0001**	**<0.001**	0.366
PDGF-bb	288 (63.8, 598)	164 (*0*, 419)	*0 (0, 0)*	**<0.001**	**0.001**	0.378
*Growth, angiogenetic, and wound healing factors*						
GM-CSF	*0 (0,* 169)	*0 (0,* 98.7)	*0 (0, 0)*	**0.036**	0.067	0.639
G-CSF	513 (68.8, 1.76∗10^3^)	195 (5.37, 999)	*0* (*0*, 435)	**0.004**	**0.034**	0.258
MCP-1	2.13∗10^3^ (1.14∗10^3^, 5.45∗10^3^)	1.48∗10^3^ (1.05∗10^3^, 2.41∗10^3^)	958 (667, 1.34∗10^3^)	**<0.001**	**0.009**	0.151
VEGF	1.13∗10^3^ (394, 1.78∗10^3^)	908 (246, 1.85∗10^3^)	1.29∗10^3^ (828, 1.68∗10^3^)	0.746	0.469	0.637
FGF basic	38.2 *(2.87*, 80.6)	27.6 *(0.67*, 60.4)	*0* (*0*, 57.5)	0.066	0.245	0.333

pOT: primary OT; rOT: recurrent OT, Mdn: median, IQR: interquartile range: significant *p* values are shown in bold; italic values are below LLOQ as defined by the manufacturer.

**Table 4:** All five cytokine ratios (IFN-*γ*/IL-4, IFN-*γ*/IL-10, IL-17/IFN-*γ*, TNF-*α*/IL-10, and IL12p70/IL-10) indicate a dominant T_h_1-type immune response.

	Control Mdn	pOT Mdn	rOT Mdn	pOT vs control *p* value	rOT vs control *p* value	pOT vs rOT *p* value
IFN-*γ*/IL-4	1	29.2	18.1	**<0.001**	**0.004**	0.212
IFN-*γ*/IL-10	0.39	16.7	6.55	**<0.0001**	**<0.001**	0.405
IL-17/IFN-*γ*	1	0.11	0.24	**<0.001**	**0.002**	0.499
TNF-*α*/IL-10	0.33	0.93	1	**0.004**	**0.005**	0.783
IL-12p70/IL-10	29.1	3.72	5.44	**0.004**	**0.031**	0.291

OT: ocular toxoplasmosis; Mdn: Median: significant *p* values are shown in bold.

## Data Availability

Raw data were generated at Charité, Augenklinik. Derived data supporting the findings of this study are available from the corresponding author (UP) upon request.

## Supplementary Materials

Supplementary Materials Supplementary Figure 1: boxplots showing the concentrations of T cell development-promoting cytokine IL-15, the T_h_2 cytokine IL-13, the T_h_17 cytokine IL-1R*α*, and the T_h_17 cell development-promoting cytokine IL-1*β* in aqueous humor of patients with pOT and rOT and the control group (*n* = 62). Supplementary Figure 2: boxplots demonstrating the concentrations of the chemokines MIP-1*α* and MIP-1*β* and the growth factors GM-CSF and G-CSF in aqueous humor of patients with pOT and rOT and the control group (*n* = 62). Supplementary Figure 3: boxplots showing the concentrations of the chemokines PDGF-bb and RANTES, the growth factor MCP-1, and angiogenetic factor VEGF in aqueous humor of patients with pOT and rOT and the control group (*n* = 62). Supplementary Table 1: correlation between immune mediator concentration and number of recurrences from both cohorts pOT and rOT (*n* = 51).
