# Supplementary material for: Immune Mediator Profile in Aqueous Humor Differs in Patients with Primary Acquired Ocular Toxoplasmosis and Recurrent Acute Ocular Toxoplasmosis
Source: Mediators Inflamm. 2019 Feb 17;2019:9356728. doi: 10.1155/2019/9356728 (PMC6398019; doi:10.1155/2019/9356728)
Supplement: Supplementary Materials — Supplementary Figure 1: boxplots showing the concentrations of T cell development-promoting cytokine IL-15, the Th2 cytokine IL-13, the Th17 cytokine IL-1Rα, and the Th17 cell development-promoting cytokine IL-1β in aqueous humor of patients with pOT and rOT and the control group (n = 62). Supplementary Figure 2: boxplots demonstrating the concentrations of the chemokines MIP-1α and MIP-1β and the growth factors GM-CSF and G-CSF in aqueous humor of patients with pOT and rOT and the control group (n = 62). Supplementary Figure 3: boxplots showing the concentrations of the chemokines PDGF-bb and RANTES, the growth factor MCP-1, and angiogenetic factor VEGF in aqueous humor of patients with pOT and rOT and the control group (n = 62). Supplementary Table 1: correlation between immune mediator concentration and number of recurrences from both cohorts pOT and rOT (n = 51). [file 9356728.f1.zip › Supplementary Figures 1-3 with legend final.pdf]

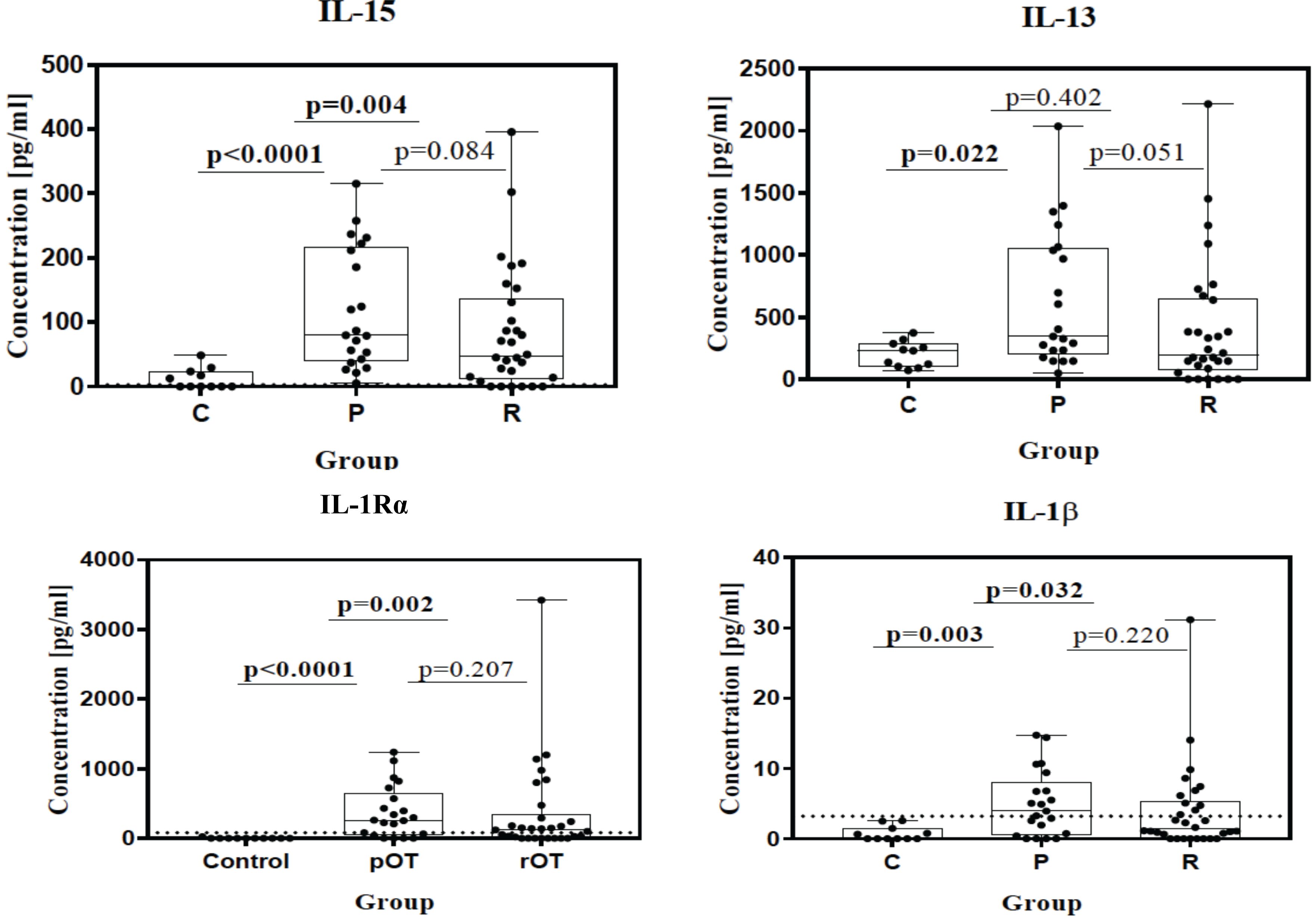

Supplementary Figure 1. Boxplots showing the concentrations of T cell development promoting cytokine IL-15, the T_h2 cytokine IL-13, the T_h17 cytokine IL-1Rα and the T_h17 cell development promoting cytokine IL-1β in aqueous humor of patients with pOT, rOT and the control group (n=62).

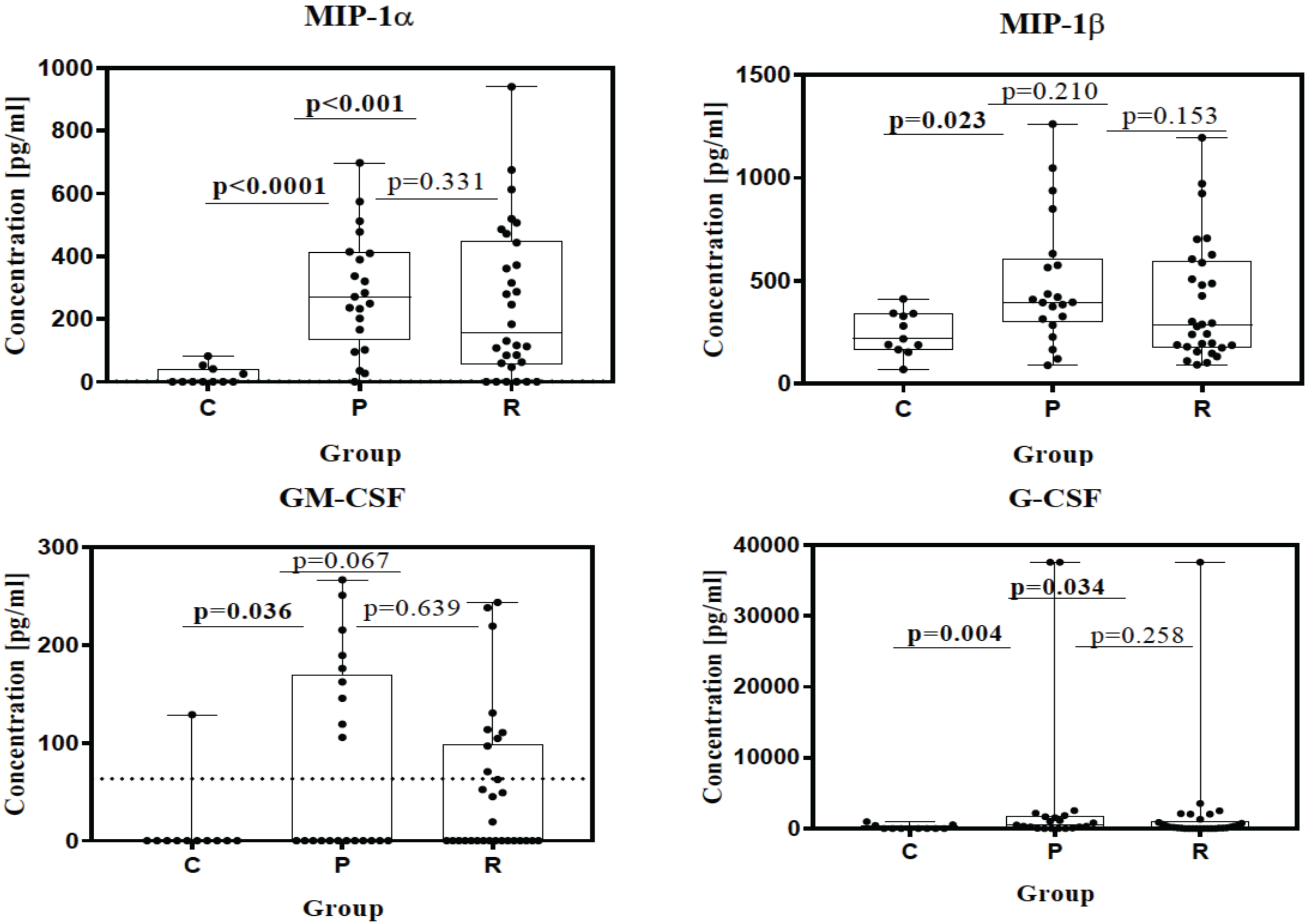

Supplementary Figure 2. Boxplots demonstrating the concentrations of the chemokines MIP-1α and MIP-1β, the growth factors GM-CSF and G-CSF in aqueous humor of patients with pOT, rOT and the control group (n=62).

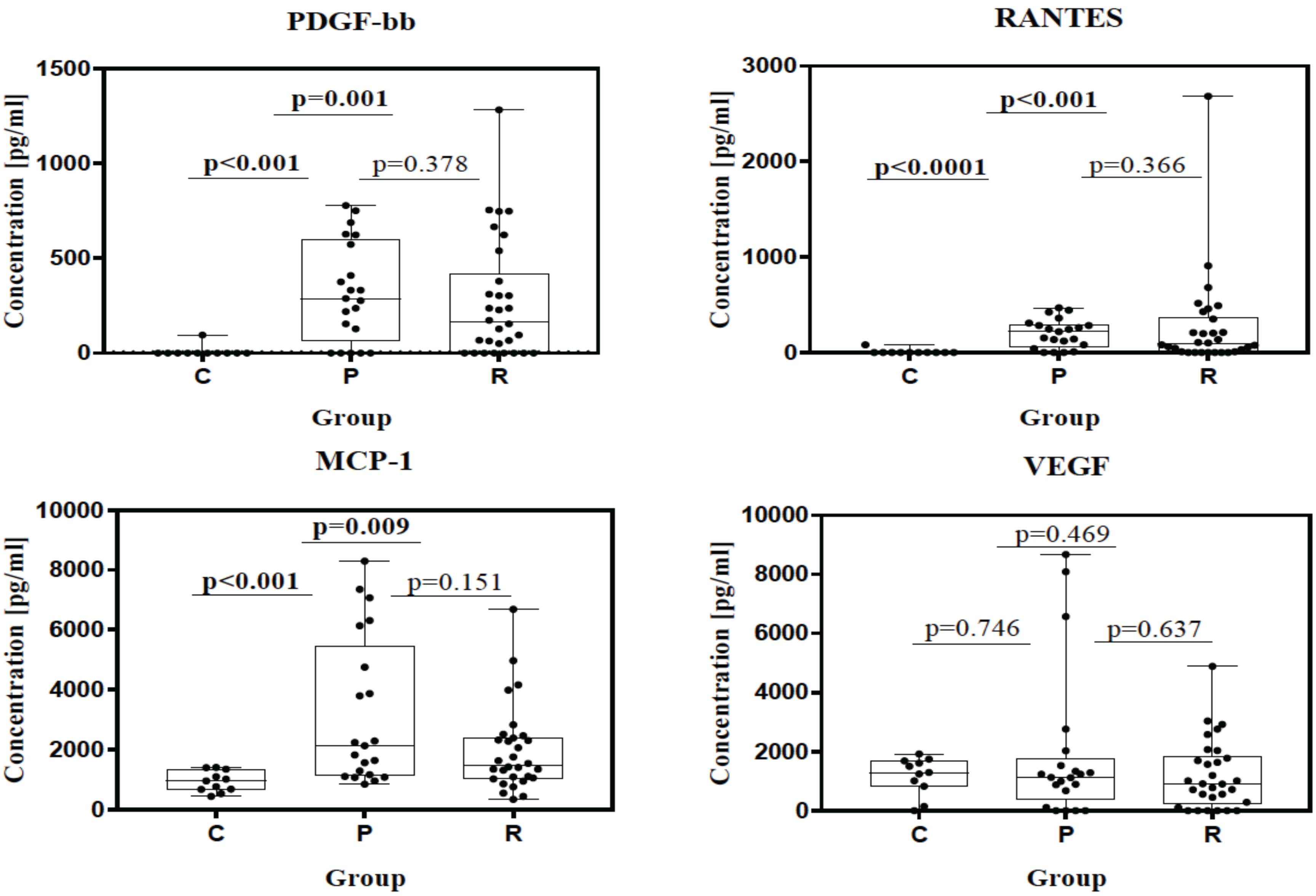

Supplementary Figure 3. Boxplots showing the concentrations of the chemokines PDGF-bb and Rantes, the growth factor MCP-1 and angiogenetic factor VEGF in aqueous humor of patients with pOT, rOT and the control group (n=62).
