# Supplementary material for: Immune Mediator Profile in Aqueous Humor Differs in Patients with Primary Acquired Ocular Toxoplasmosis and Recurrent Acute Ocular Toxoplasmosis
Source: Mediators Inflamm. 2019 Feb 17;2019:9356728. doi: 10.1155/2019/9356728 (PMC6398019; doi:10.1155/2019/9356728)
Supplement: Supplementary Materials — Supplementary Figure 1: boxplots showing the concentrations of T cell development-promoting cytokine IL-15, the Th2 cytokine IL-13, the Th17 cytokine IL-1Rα, and the Th17 cell development-promoting cytokine IL-1β in aqueous humor of patients with pOT and rOT and the control group (n = 62). Supplementary Figure 2: boxplots demonstrating the concentrations of the chemokines MIP-1α and MIP-1β and the growth factors GM-CSF and G-CSF in aqueous humor of patients with pOT and rOT and the control group (n = 62). Supplementary Figure 3: boxplots showing the concentrations of the chemokines PDGF-bb and RANTES, the growth factor MCP-1, and angiogenetic factor VEGF in aqueous humor of patients with pOT and rOT and the control group (n = 62). Supplementary Table 1: correlation between immune mediator concentration and number of recurrences from both cohorts pOT and rOT (n = 51). [file 9356728.f1.zip › Supplementary Table 1 for submission final.pdf]

Supplementary Table 1. Correlation immune mediator concentration and number of recurrences from both cohorts pOT and rOT (n=51)

Immune mediator	p value
IL-1β	0.100
IL-1Rα	**0.045**
IL-2	0.159
IL-4	0.064
IL-5	0.096
IL-6	**0.045**
IL-7	**0.017**
IL-8	**0.047**
IL-9	**0.008**
IL-10	0.147
IL-12p70	0.238
IL-13	**0.024**
IL-15	**0.042**
IL-17	0.188
Eotaxin	0.092
FGFbasic	0.079
G-CSF	0.094
GM-CSF	0.538
IFN-γ	**0.024**
IP-10	0.059
MCP-1	0.102
MIP-1α	0.094
PDGF-bb	0.123
MIP-1β	0.109
RANTES	0.092
TNF-α	**0.019**
VEGF	0.616

[a]significant p-values are shown in bold
